# Phenotype-aware prioritisation of rare Mendelian disease variants

**DOI:** 10.1016/j.tig.2022.07.002

**Published:** 2022-08-04

**Authors:** Catherine Kelly, Anita Szabo, Nikolas Pontikos, Gavin Arno, Peter N. Robinson, Jules O.B. Jacobsen, Damian Smedley, Valentina Cipriani

**Affiliations:** 1 William Harvey Research Institute, Queen Mary University of London, London, EC1M 6BQ, UK; 2 UCL Institute of Ophthalmology, University College London, London, EC1V 9EL, UK; 3 The Jackson Laboratory for Genomic Medicine, Farmington, CT 06032, USA; 4 Moorfields Eye Hospital NHS Foundation Trust, London, EC1V 2PD, UK; 5 UCL Genetics Institute, University College London, London, WC1E 6AA, UK

## Abstract

A molecular diagnosis from the analysis of sequencing data in rare Mendelian diseases has a huge impact on the management of patients and their families. Numerous patient phenotype-aware variant prioritisation (VP) tools have been developed to help automate this process, and shorten the diagnostic odyssey, but performance statistics on real patient data are limited. Here we identify, assess, and compare the performance of all up-to-date, freely available, and programmatically accessible tools using a whole-exome, retinal disease dataset from 134 individuals with a molecular diagnosis. All tools were able to identify around two-thirds of the genetic diagnoses as the top-ranked candidate, with LIRICAL performing best overall. Finally, we discuss the challenges to overcome most cases remaining undiagnosed after current, state-of-the-art practices.

## Molecular diagnosis in rare Mendelian diseases using phenotype-aware VP software tools

With approximately 80% of rare diseases having a genetic origin, identifying the correct causative variants in rare Mendelian single-gene disorders creates a greater potential for informed clinical management through precision medicine or recommendation for drug trials, rather than only treating evident symptoms. Improvements in sequencing genetic information at scale through parallelisation (next-generation sequencing) have enabled greatly increased quantities of genomic data production at lower overall costs, as shown by the recent completion of the 100,000 Genomes Project in the UK [1]. Whole-exome sequencing (WES) is still the most commonly used method, as the exome (~2% of the human genome) harbours ~85% of currently known disease-causing sequence variants [2]. The candidate variants from a typical WES experiment are often derived from 60 000 to 100 000 variants affecting protein-coding regions, of which nearly all will be benign or unrelated to the disease [3]. However, the filtering and review process can still involve many tens, if not a few hundreds, of candidate variants and is usually both time-consuming and expensive if done via manual analysis by multidisciplinary clinicians and scientists. Around one-third of children born with rare genetic diseases do not live to see their fifth birthday [4], so it is vital that their molecular diagnosis is rapid and yet, the traumatic wait time for patients is often lengthy (e.g., a median of 6 years in the 100,000 Genomes Project) [1]. VP software offers the possibility of identifying the correct disease-causative variants more efficiently, sometimes within minutes. These tools usually discard large quantities of likely benign, common variants through filtering strategies based on publicly available (e.g., gnomAD) and in-house sequencing databases.

The vast majority of VP tools are still only able to prioritise single-nucleotide variants (SNVs) and small insertion/deletions (indels) formatted as variant call format (VCF) files. To determine likely rare disease-causative SNVs/indels, VP tools usually incorporate several existing *in silico* pathogenicity prediction tools that can restrict the patients’ VCF files to variants of interest based on a range of methods. They include function-prediction methods (e.g., MutationTaster, PolyPhen-2, SIFT), which are based on the likelihood of each missense variant causing pathogenic changes to protein structure or function; phylogenetic conservation methods (e.g., GERP++, phastCons, phyloP), which measure the degree of conservation at a given nucleotide site; other more recent methods, which concern a tailored use of deep neural networks (e.g., MVP, PrimateAI); and ensemble methods (e.g., CADD, DANN, REVEL), which integrate information from multiple component methods [5]. Despite the availability of this wide range of *in silico* pathogenicity prediction tools, improvements are still needed to discriminate pathogenic from benign variants with a reported median specificity of 65%; furthermore, with sensitivities ranging from 51% to 96% (median, 88%), relying only on algorithm-predicted variant pathogenicity is known to still generate a large number of false positive candidates [5].

With the aim of automating the manual prioritisation of candidate variants made by clinicians and scientists where the relevance of a certain gene variant to a patient’s phenotype is taken into account, virtually all recent VP software tools have now enabled the incorporation of standardised patients’ phenotypic terms, drawing from the more than 15 000 terms of the Human Phenotype Ontology (HPO) [6]. This has ultimately been a significant addition; for example, Exomiser (among the first VP tools of its kind) [7,8] demonstrated an increased top prioritisation of the correct diagnosed causative variants from 20–77% (using only variant-based filtering) to 96–97% (with the addition of patients’ HPO terms) using simulated sequencing data and across different mode of inheritances (MOIs) as well as from 3% to 74% using real patient data and inferred MOIs [9].

The VP software tools to date have been tested on (different) simulated and/or very small real patient sequencing datasets, with limited software performance comparison. Strikingly, each specific published tool virtually always claims to outperform the relatively limited number of other tools tested from the literature. Here, we set out to perform a thorough literature review with the aim of identifying up-to-date phenotype-aware VP software tools. Building on a previous benchmarking of VP tool Exomiser [9], we then conducted a relatively unbiased software performance comparison of the selected VP tools using a dataset of 134 whole-exomes from individuals affected by a range of rare inherited retinal diseases (IRDs) and known molecular diagnosis.

## Up-to-date phenotype-aware VP software tools

A detailed literature search was carried out to determine a list of phenotype-aware VP software tools to use for real patient data benchmarking that would meet the following criteria: (i) directly accepting sequencing data formatted as VCF files; (ii) accepting HPO terms to describe patients’ phenotypes; (iii) being relatively up-to-date (last updated or published since 2018); (iv) freely available for academic use; and (v) with local, programmatic access (and therefore safer for use with patient data as opposed to web-based access and allowing processing of data at scale).

**Figure 1.**
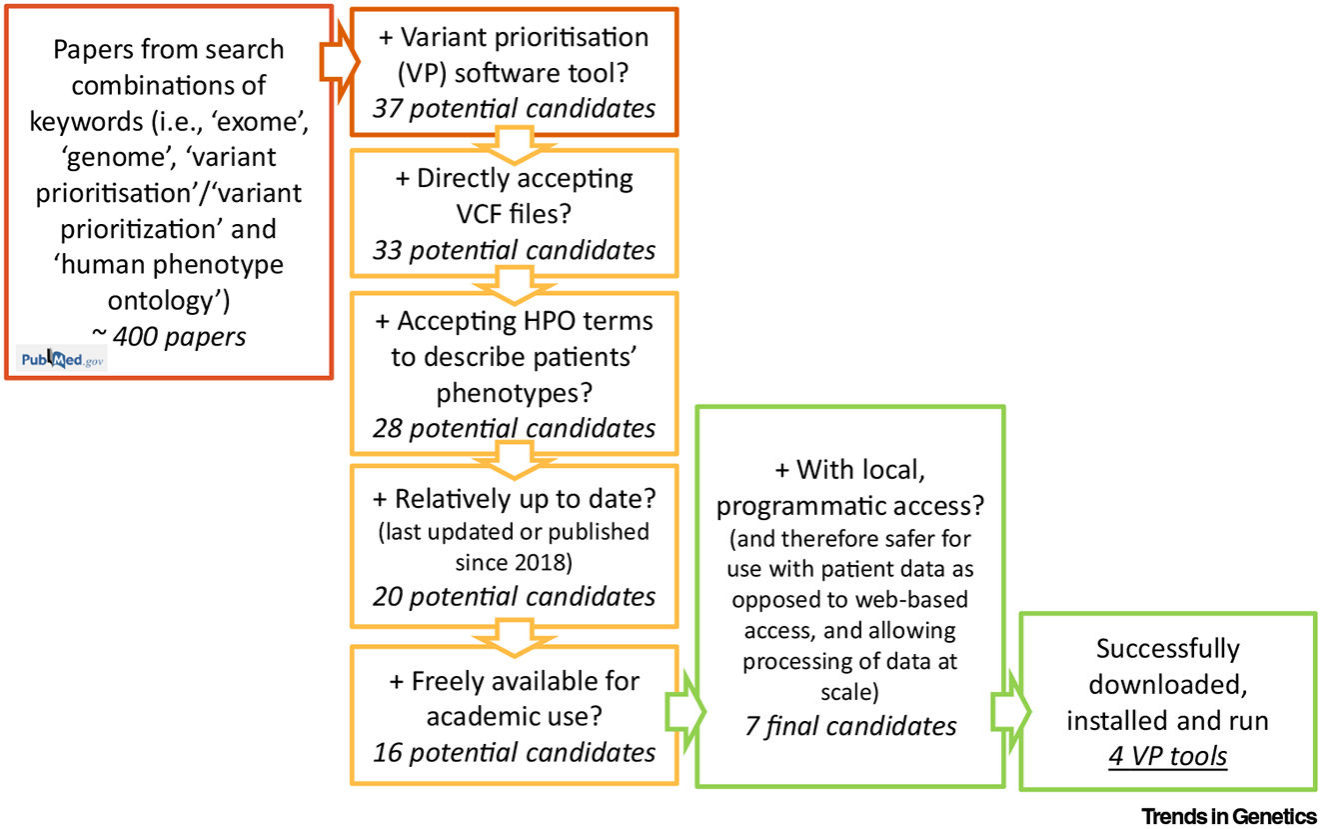
Flow chart of the literature search and filtering criteria used to select the final phenotype-aware variant prioritisation (VP) software tool candidates for benchmarking on real patient data. Searches of combinations of keywords (red box) were conducted in PubMed. Phenotype-aware VP software tool candidates found in the resulting papers (37) were then narrowed down to seven final candidates based on five criteria: accepting variant call format (VCF) files; accepting Human Phenotype Ontology (HPO) terms; last updated or published since 2018; freely available; with local, programmatic access. Finally, four VP tools were successfully downloaded, installed, and run on real patient data for testing and comparison.

Literature searches using a combination of keywords (i.e., ‘exome’, ‘genome’, ‘variant prioritisation’, and alternative spelling ‘variant prioritization’, and ‘human phenotype ontology’) were conducted in PubMed and returned about 400 peer-reviewed journal articles (11 March 2022) (Figure 1). Articles were screened to identify those publications that involved a VP software tool for rare Mendelian disease. This initially gave a list of 37 candidate VP software tools [3,8,10-44] to prune according to the aforementioned criteria. Remarkably, only seven VP software tools passed all five criteria and were selected as final candidates for testing and comparison. Table 1 shows the details of the 37 tools retrieved from the literature search and the corresponding selection process.

Most of the tools (33) can directly accept sequencing data as a VCF file, which is the standard file format for storing genetic variation data. A total of 28 tools are ‘phenotype-aware’ as opposed to simply using the genetic variant data for prioritisation; they all allow an integrative analysis of the patients’ phenotypes using the HPO, which has become the *de facto* standard for deep phenotyping in the field of rare disease [6]. The most discriminating criterion (failed by 23 tools) is our requirement for the VP tool to provide both local and programmatic access. Local installation is usually essential to conform to patient data privacy and security rules. Also, despite some attractive features web-based tools may seem to provide, processing of data at scale via programmatic access is usually vital to guarantee efficient analysis pipelines. It has to also be noted that 11 tools were never updated since their publication date or 2017 (one in 2013, two in 2014, three in 2015, one in 2016, and four in 2017), with corresponding website link broken for one of them (Table 1). This is largely a reflection of the challenges of maintaining academic software when resources do not exist for such an activity. Finally, Table 2 shows a summary of the different data sources that are leveraged within each of the seven remaining VP tool candidates to document the type and amount of information each tool relies on, as well as to provide insights into the need to update and/or maintain them.

## Four VP software tools successfully downloaded, installed, and run using real patient data

Attempts were made to download and install all seven of the selected VP software tools. Further illustrating the problems with long-term maintenance of academic software, this was not possible for three of them due to inaccessible databases, failing dockers, or lack of information in ReadMe files. In particular, for DeepPVP [16], we were unable to follow their installation process as no phenomenet-vp docker container exists in Docker Hub and the dockerfile recipe provided in their GitHub repository does not build in the research computing environment containing our clinical data; Phenoxome’s [42] docker pull was successful but there were no further instructions for progression with its installation; VARPP [11] download of the required dbNSFP database (version v3.4a) was no longer possible. The four remaining VP software tools were finally included in this software performance evaluation and comparison using real patient data as they were each successfully downloaded and installed as reported next, including a brief description of the corresponding VP rationale and algorithms.

The corresponding code for all analyses is available as a repository at https://github.com/whri-phenogenomics/VPSoftware_review.

### Exomiser

Exomiser [7-9,45] is a freely available Java software tool that automates filtering and prioritisation of variants contained in VCF files from sequencing of rare disease patients (and, if available, their family members). A range of user-defined variant filtering criteria can be applied based on JANNOVAR [46] functional annotation, minor allele frequency, and expected inheritance pattern, amongst others. Each filtered variant is then prioritised according to a variant score based on its rarity and *in silico* algorithm-predicted pathogenicity, which is in turn combined with a corresponding gene-specific phenotype score. The latter is obtained via the PhenoDigm algorithm [37] and is calculated based on the semantic similarity between the user-provided HPO-encoded patient’s phenotype and the phenotypic annotations of genes in known human diseases, orthologs in mouse and zebrafish model organisms, and phenotypes of protein–protein associated neighbours [7].

The download and installation of Exomiser version 13.0.0 (released on 23 September 2021) were straightforward, following a comprehensive ReadMe file accessed via Exomiser’s GitHub page^i^. We used a Bash script to create a single-sample-analysis-settings.yml file starting from the preset-exome-analysis.yml example file provided and containing the Exomiser analysis settings per each patient from the IRD dataset. Exomiser was then run using the following command line per each single-sample analysis for the IRD patient WES dataset (Java version 17.0.0; Exomiser variant and phenotype databases version 2109; default Ensembl transcript annotation):

java -Xms2g -Xmx4g -jar exomiser-cli-13.0.0.jar -analysis single-sample-analysis-settings.yml

A few representative sections of the HTML output file from the analysis of one single sample are reported in Figure S1 in the supplemental information online. Tab-separated (tsv) output files containing a variety of relevant information for the filtered and prioritised variants (including functional annotation, allele frequency in publicly available databases, the gene-specific phenotype score, the variant score, and the Exomiser combined score) were also obtained and processed for software performance evaluation and statistical comparison, as described later and in the supplemental information online.

### PhenIX

PhenIX (i.e., phenotypic interpretation of exomes) [44] is a computational method that evaluates and ranks variants based on their rarity and predicted pathogenicity, as well as the semantic similarity of the HPO terms used to describe the patients' phenotypes to those of thousands of human Mendelian diseases as reported in OMIM and Orphanet (last updated in 2019).

PhenIX is available within Exomiser. Therefore, it did not require any additional download and installation, can be run in the same way as Exomiser, and produces similar output files.

It exploits the same variant filtering framework of Exomiser, while its semantic similarity algorithm is enabled by replacing Exomiser option ‘hiPhivePrioritiser: {}’ with ‘phenixPrioritiser: {}’.

### LIRICAL

LIRICAL (i.e., likelihood ratio interpretation of clinical abnormalities) [35] exploits the likelihood ratio (LR) statistical framework. Not only does it ultimately rank the candidate variants but it also provides an estimate of the post-test probability of candidate diagnoses and calculates the extent to which (LR) each provided HPO-encoded abnormality (and, if VCF files are available, genotype too) is consistent with the diagnosis.

LIRICAL version 1.3.4 (released on 26 September 2021) was downloaded by git cloning the corresponding GitHub repository^ii^ and installed following the clear instructions from the corresponding ‘readthedocs’ pages^iii^. LIRICAL makes use of the Exomiser variant and phenotype databases (we enabled database version 2109). The preferred input format for LIRICAL is Phenopackets^iv^, an open standard, also adopted within the Global Alliance for Genomics and Health^v^, for sharing detailed phenotypic descriptions linked with disease, patient, and genetic information.

We used a Python script to create a Phenopacket single-sample-phenopacket.json per each patient from the IRD patient WES dataset. LIRICAL was then run using the following command line per each single-sample analysis:

java -jar LIRICAL.jar phenopacket -p single-sample-phenopacket.json-e *path/to/Exomiser-data-directory* -x *prefixOfOutputFile* -tsv -output-directory *path/to/output-directory*

A few representative sections of the HTML output file from the analysis of one single sample are reported in Figure S1 in the supplemental information online. Tab-separated (tsv) output files containing relevant information for the candidate prioritised diagnoses, together with the corresponding filtered variants (including rank, post-test probability, and LR), were also obtained and processed for software performance evaluation and statistical comparison, as described later and in the supplemental information online.

### Xrare

Xrare [30] concerns a newly developed phenotypic similarity measure called emission-reception information content (ERIC), which is claimed to be somehow robust to imprecise and noisy clinical phenotypes and to be a machine learning approach (i.e., a gradient boosting decision tree algorithm implemented in XGBoost [47]) that can jointly model phenotypic features and multiple genetic features, including American College of Medical Genetics and Genomics/Association for Molecular Pathology (ACMG/AMP) guideline-based features for VP.

Xrare was downloaded and installed converting the Docker image xrare-pub-2015.docker.tar.gz^vi^ into a Singularity container (xrare-2015.simg) due to preferences of the Queen Mary University (QMUL) Apocrita high-performance computing (HPC) facility.

Per each single-sample analysis in the IRD patient WES dataset, we first ran the Xrare module via singularity run xrare-2015.simg and then an R script with core code consisting of the command xrare() with arguments vcffile and hpoid (i.e., a single-sample gzipped VCF file and a corresponding string of HPO terms, e.g., as ‘HP:0001156, HP:0001363, HP:0010055’), respectively.

If xrare(), not being a robust function, did not halt execution due to ‘system run_annotation is failed’ errors, tsv output files containing a variety of relevant information for the filtered and prioritised variants (including functional annotation, allele frequency in publicly available databases, many pathogenicity prediction variant scores, the ACMG/AMP-based classification, and the Xrare score) were produced and processed for software performance evaluation and statistical comparison, as described later and in the supplemental information online.

## Real patient WES dataset with known molecular diagnosis and HPO-encoded clinical diagnosis

We assessed the performance of the four successfully downloaded and installed VP software tools using the same real patient WES dataset that was previously described and used in a benchmarking of Exomiser [9]. Briefly, the dataset consists of 134 individuals who had been clinically diagnosed with IRD by a consultant ophthalmologist at Moorfields Eye Hospital and the University College London Institute of Ophthalmology (London, UK) and had also received a molecular diagnosis (i.e., ‘solved’) based on SNVs and/or indels from the analysis of their respective WES data (Tables S1 and S2 in the supplemental information online). All patients had been sequenced as singletons. For this review and software performance comparison analysis, we used a more recent version of single-sample VCF files than the ones used previously [9], which were obtained as described in the supplemental information online. As to the phenotypic information, each of the 19 clinical diagnoses observed in the real patient WES dataset had been assigned a parsimonious, fixed list of most representative HPO terms (from one single term to six terms) by three ophthalmologists with expertise in IRD diagnosis (Table S1 in the supplemental information online). Encoding the patient’s clinical diagnoses into HPO terms can overcome the need for single patient-specific HPO terms and has already proven to be effective [9]; however, many tools now exist that facilitate an efficient collection of patients’ HPO-encoded clinical phenotypes and/or can automate the extraction of human disease phenotypes from free text clinical notes as well as electronic health records [48-52]; also, large-scale sequencing projects now require the availability of patients’ phenotypes encoded as HPO terms as study requirements [1]. Evaluations based on patient-specific HPO terms would additionally be affected by how specific and extensive these terms were (annotation sufficiency) and how thorough the reference disease annotations are in terms of covering both common and rarely observed signs and symptoms.

### Software performance evaluation and statistical comparison using the IRD patient WES dataset

Each selected VP software tool was run on each of the 134 whole-exomes from the IRD dataset using default commands as described in the available documentation. Also, to perform a fairly unbiased software performance comparison, the corresponding output was processed in a way that would mimic IRD diagnostics and produce a relatively homogeneous set of criteria across tools. Finally, we noted the rank at which each VP tool outputted the known diagnosed disease variant(s) per each IRD patient and ran a set of statistical tests to assess pairwise agreement. More details are provided in the supplemental information online.

**Figure 2.**
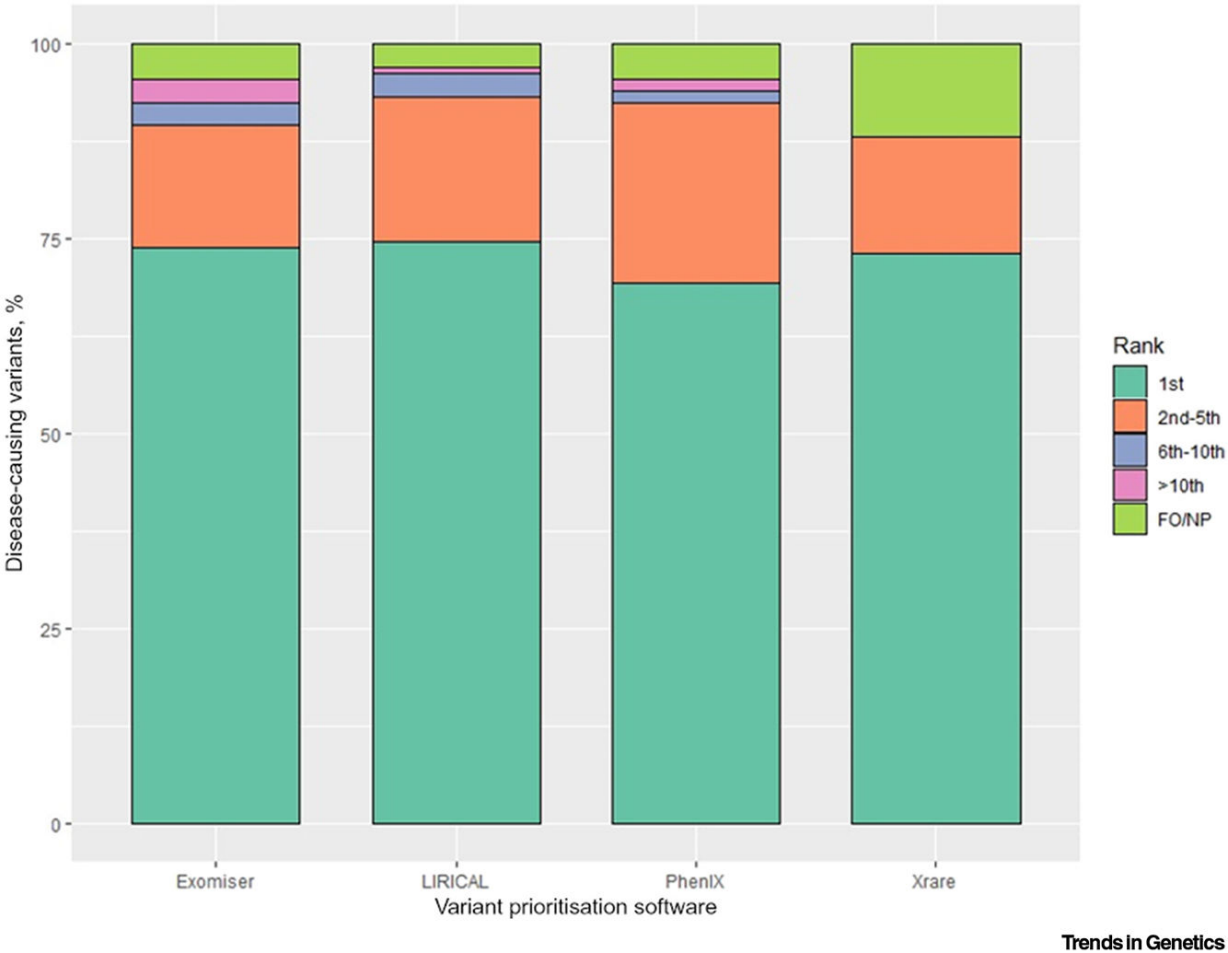
Bar plot of the percentage categorical distribution of the disease-causing variant ranking in the inherited retinal disease (IRD) patient whole-exome sequencing (WES) dataset for the four successfully tested phenotype-aware variant prioritisation (VP) software tools. The ranking results were categorised into five mutually exclusive bins: ‘Top’ (including top ties), ‘(2–5)’, ‘(6–10)’, ‘>10’, and ‘Filtered out/Not prioritised’ (FO/NP) (the latter being any disease-causing variant(s) failed to be kept in during the filtering/prioritisation step).

**Figure 3.**
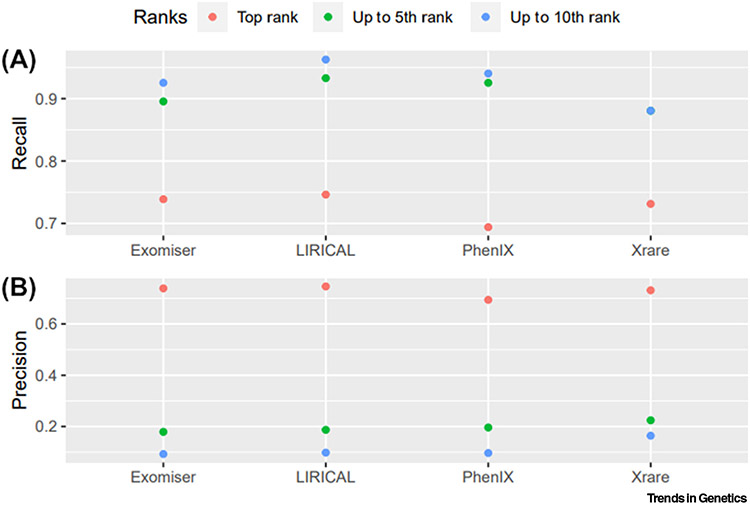
Scatter plots of diagnostic measurements for the software performance of the four successfully tested phenotype-aware variant prioritisation (VP) software tools. (A) Recall (true positive rate) and (B) precision were calculated from confusion matrices for the correctly diagnosed variants matched in the first rank, up to the fifth rank, and up to the tenth rank by each of the tested VP software tools. Recall (true positive rate) = true positives (TP)/actual positives; precision = TP/predicted positives. For Xrare, the recall estimate for the ‘up to the fifth rank’ analysis is equal to the recall estimate for the ‘up to the tenth rank’ analysis (i.e., 0.88).

LIRICAL, Exomiser, and Xrare performed similarly at assigning the correct causative variants first rank for about two-thirds of the patients (100, 99, 98, respectively, out of 134) (Table 3, Figures 2 and 3A, and Figure S2 in the supplemental information online), while PhenIX performed the worst with 93 patients. The known diagnosed variants were ranked top 5 in about 93% of the dataset by LIRICAL and PhenIX and about 90% by Exomiser, while Xrare remained at 88% for both top 5 and top 10 ranking categories. LIRICAL showed the best performance also at assigning the correct causative variants top 10 ranks for over 96% of the dataset, closely followed by PhenIX and Exomiser. We also calculated precision estimates from statistical confusion matrices for the correctly diagnosed variants matched in the first rank, up to the fifth rank, and up to the tenth rank by each of the tested VP software tools (Figure 3B). For example, should a clinical geneticist review the top five candidates, with precision estimates equal to about 0.2 for all four VP tools, one in five reported variants would be the correct ones. These results are similar to those reported in previous studies on other clinical cohorts where diagnoses were detected as the top ranking candidate in 60% of cases for LIRICAL [35], 77% for Exomiser [1], ~40% and ~80% for Xrare [30], whilst PhenIX reported a mean rank of 2.1 [44]. A further evaluation of several tools on two clinical datasets showed performances of 41–51%, 15–26%, 16–43%, and 38–51% of diagnoses identified as the top-ranked candidate for LIRICAL, Exomiser, PhenIX, and Xrare, respectively [53], but the settings for Exomiser and PhenIX differed markedly from those used here in terms of pathogenicity prediction algorithms as well as frequency and non-PASS variant filtering [58].

All VP software tools showed the same median rank equal to 1, with Exomiser presenting with the highest maximum rank (123), while Xrare demonstrated the smallest one (5.5). Importantly, the latter is based on Xrare having missed (‘Filtered out/Not prioritised) 16 correct diagnoses out of 134. That is the highest number overall, with Exomiser and PhenIX having missed six each and LIRICAL the lowest number of four. In particular, the latter four genetic diagnoses were missed by all VP tools due to the corresponding variants being flagged as low quality in the VCF file (three) or not called at all due to low coverage (one). Additionally, Exomiser and PhenIX chose to prioritise different variants for the correct gene other than the correct ones for two samples each, and Xrare either only partially outputted/did not output at all 11 diagnoses (seven homozygous, four compound-heterozygous) or missed one diagnosis due a genotype mismatch in the VCF file (the latter was rescued by Exomiser and PhenIX as a ClinVar^vii^, ‘whitelisted’ variant).

Finally, taking LIRICAL as the reference VP tool given that it showed the best performance estimates overall (Table 3, Figures 2 and 3, and Figure S2 in the supplemental information online), we used statistical tests which are tailored to assess agreement between two raters to gain insights into whether any VP tool performed significantly better (Table 3). The agreement with LIRICAL’s performance (i.e., same disease-causing variant ranked same by the two VP tools) did not exceed 70% for any of the other three tested VP tools (i.e., 69%, 67%, and 62% for Exomiser, PhenIX, and Xrare, respectively); corresponding ‘fair’, ‘fair’, and ‘slight’ agreements were observed when calculating the Cohen’s kappa values (interpreted according to Landis and Koch’s guidelines, i.e., kappa < 0.00 as ‘poor’ agreement, 0.00–0.20 as ‘slight’, 0.21–0.40 as ‘fair’, 0.41–0.60 as ‘moderate’, 0.61–0.80 as ‘substantial’, and 0.81–1 as ‘almost perfect’ agreement [54]) (Table 3). However, these estimates represent a statistically significant difference only when comparing LIRICAL’s performance with the overall worse performance of Xrare (Stuart-Maxwell test, *P* = 1.6 × 10^−3^). Interestingly, despite LIRICAL performing best overall, we still observed a relevant number of molecular diagnoses that were better ranked by the second tool (i.e., 20, 19, 24 for Exomiser, PhenIX, and Xrare, respectively) (Table 3).

## Concluding remarks

Despite numerous publications in the past decade showing the promise of phenotype-aware, VP tools for Mendelian diseases, very few are actually available for up-to-date analysis and integration into local diagnostic pipelines. Notably, although most tools are freely available, they are often developed as web-/app-based access only, without the possibility of being installed locally (Table 1). Both those features present major disadvantages. If the lack of programmatic access for a certain tool should be seen as impractical, especially in large-scale analyses, for example, making a user upload many data files manually on a website, the impossibility of installing a tool locally severely limits the usability of the tool itself due to strict privacy and security requirements of most, if not all, real patient datasets, including the IRD patient WES dataset tested in this review. Guaranteeing local tool installation, either with programmatic or web-/app-based access, should be considered essential when developing VP software. However, all four tools benchmarked on real patient data in this review showed an impressive capacity to prioritise diagnoses and lighten the load of clinical geneticists (Table 3 and Figure 2). Each is likely to offer its own advantages and disadvantages (e.g., LIRICAL offers an interpretable graphical overview of the evidence based on LRs; Figure S1 in the supplemental information online), but it is likely that the use of several different approaches will minimise the chance of overlooking a diagnosis.

Each of the assessed tools uses similar approaches to variant filtering and prioritisation, with most of the algorithmic differences being in how phenotypic data were used in this process. The variant data sources do vary considerably between each tool though (Table 2); this could account for performance differences and it is hard to separate it out from any differences due to the phenotype prioritisation algorithm. However, Exomiser and PhenIX used exactly the same framework and data for variant annotation, filtering, and prioritisation, so the reduced performance of PhenIX is due to its human-only, semantic phenotypic similarity approach relative to Exomiser’s multispecies algorithm. PhenIX’s underlying reference data have not been updated since 2019, so this could also explain much of the observed difference. LIRICAL and Exomiser share much of the same data and variant annotation and filtering approaches, so the improved performance observed for LIRICAL could well be due to its LR statistical approach. Here, Xrare’s use of emission-reception information content and XGBoost machine learning approach showed a similar performance to the other tools but may well show improved performance on other real clinical datasets where imprecise and noisy phenotype annotations have been collected.

Several questions remain to be addressed (see Outstanding questions). The key challenge for the future is how to tackle the overwhelming numbers of cases that remain undiagnosed even after state-of-the-art data analysis. For example, 75% of cases were undiagnosed in the 100,000 Genomes Project after whole-genome sequencing and a combination of gene-panel, Exomiser, and research analysis [1]. About half of the nearly one million variants reported in ClinVar as associated with severe genetic diseases are indeed of uncertain significance (VUS) or present conflicting annotations. This exposes how the newly emerging functional variant assays, ideally to deploy at scale, are likely to attract substantial efforts in rare disease diagnostics to assess the exact impact of associated variants on gene function [55]. Part of the uncertainty around disease-associated variants is also likely to be resolved by the ever-increasing availability of sequencing data as well as global data sharing initiatives that connect databases of genomic and phenotypic data (e.g., Matchmaker Exchange) [56,57]. Yet, this inevitably poses additional challenges, as patient data privacy and security remain paramount. Despite the current challenges, many new disease–gene associations are being published every month and VP tools, provided they are reasonably up-to-date, offer a convenient way to help reinterpret unsolved cases by automated reanalysis and identification of new candidates. Finally, a large proportion of unsolved rare disease cases are likely to involve noncoding and structural variation that is being overlooked by the current sequencing and analysis approaches. Although some progress has been made in VP tools offering analysis of these types of variation, algorithm improvements are going to be needed before validation and diagnosis of these become feasible at scale.

## Supplementary Material

Supplementary material

## Figures and Tables

**Table 1.** Selection of phenotype-aware variant prioritisation (VP) software tools based on five suitability criteria ^a^

VP software tool	Directly accepting VCF files	Accepting HPO terms	Last updated or published since 2018	Freely available	Local, programmatic access	Refs
Exomiser^b^	√	√	√	√	√	[8]
LIRICAL^b^	√	√	√	√	√	[35]
VARPP^b^	√	√	√	√	√	[11]
Xrare^b^	√	√	√	√	√	[30]
Phenoxome^b^	√	√	√	√	√	[42]
DeepPVP^b^	√	√	√	√	√	[16]
PhenIX^b^	√	√	√	√	√	[44]
VINYL		√	√	√	√	[18]
eDiva		√	√	√	√	[15]
VarSight		√	√	√	√	[20]
AMELIE	√	√	√	√	No local installation	[14]
GeneTerpret	√	√	√	√	Web-based only	[32]
PhenoPro	√	√	√	√	Web-based only	[31]
MutationDistiller	√	√	√	√	Web-based only	[22]
GenIO	√	√	√	√	Web-based only	[28]
PhenoVar	√	√	√	√	Web-based only	[40]
GEM	√	√	√	Commercial	Web-based only	[3]
EVIDENCE	√	√	√	Commercial	Web-based only	[36]
VarElect	√	√	√	Commercial	Web-based only	[39]
Phevor (now Phevor2)		√	√	√	Web-based only	[38]
Moon	√	√	√	Commercial	Code available on request	[34]
wAnnovar	√	√		√	Web-based only	[43]
OVA	√	√		√	Web-based only	[12]
BierApp	√	√		√	Web-based only	[10]
OMIM Explorer	√	√		√	Broken web link	[25]
QueryOR	√	√		√	Web-based only	[13]
eXtasy	√	√		√	√	[37]
Phen-Gen	√	√		√	√	[26]
PVP	√	√		√	√	[17]
VCF.Filter	√			√	App-based only	[33]
wKGGSeq	√			√	Web-based only	[29]
VAAST	√			Commercial	Web-based only	[27]
VPOT	√		√	√	√	[24]
Ensembl Variant Effect Predictor	√		√	√	No local installation	[23]
Clin.iobio	√	√	√	√	Web-based only	[41]
VarFish	√	√	√	√	Web-based only	[21]
VarAFT	√	√	√	√	App-based only	[19]

a A grey cell indicates that the corresponding feature is not present.

b Following the completion of a literature review, seven viable VP software tool candidates that met all five suitability criteria (i.e., directly accepting VCF files; accepting HPO terms; last updated or published since 2018; freely available; with local, programmatic access) were selected for testing and comparison.

**Table 2.** Data sources used within each of the seven selected phenotype-aware variant prioritisation (VP) software tools from the literature review^a^

VP software	HPO (OMIM, Orphanet)	1000 Genomes	CADD	ClinVar	ExAC	UK10K	NHLBI ESP	FANTOM5	gnomAD	HGMD	MutationTaster	PolyPhen-2	RefSeq	SIFT	STRING Network	UberPheno	dbSNP	Ensembl	M-CAP	MPC	MVP	PRIMATE-AI	REVEL	TOPMed	UCSC	DANN	MP	Refs
Exomiser^b^																												[8]
LIRICAL^b^																												[35]
PhenIX^b^																												[44]
Xrare^b^																												[30]
VARPP																												[11]
Phenoxome																												[42]
DeepPVP																												[16]
Total number of software tools	7	6	6	6	6	5	5	4	4	4	4	4	4	4	4	4	3	3	3	3	3	3	3	3	3	2	2	

a ZFIN, IMPC, and MGD are used only by Exomiser. SPIDEX, Pfam, Treefam, GIANT, REACTOME, LRT, InterPro, GWAS, Blast, GO, GERP, dbscSNV, and phyloP are used only by Xrare. ARIC, GTEx, and MetaSVM are used only by VARPP. GWAVA is used only by DeepPVP.

b The tools that were successfully downloaded, installed, and tested in the software performance comparison within this study.

**Table 3.** Descriptive statistics of the disease-causing variant ranking in the inherited retinal disease (IRD) patient whole-exome sequencing (WES) dataset for the four successfully run phenotype-aware variant prioritisation (VP) software tools and pairwise agreement with LIRICAL (reference)^a^

VP software tool	Patients with disease-causing variants filtered out/not prioritised	Mean rank (SD)	Median rank	Min rank	Max rank	Top rank, % (*n* = 134)	Agreement with LIRICAL^b^ (%)	Disagreement with LIRICAL^b^(%)	Cohen's kappa (Agreement)	Stuart-Maxwell test
LIRICAL	4	1.7 (2.0)	1	1	18	74.6	Reference			
Exomiser	6	3.1 (11.7)	1	1	123	73.9	68.7	Better: 14.9 Worse: 16.4	0.25 (fair agreement)	0.4189
Xrare	16	1.4 (0.8)	1	1	5.5	73.1	61.9	Better: 17.9 Worse: 20.1	0.10 (slight agreement)	0.0016
PhenIX	6	1.8 (2.5)	1	1	23	69.4	67.2	Better: 14.2 Worse: 18.7	0.25 (fair agreement)	0.3894

a The mean/median/min/max ranks in the table refer to the effective *n*, that is, 134 minus the number of patients with disease-causing variants filtered out. ‘Top rank, %’ describes the percentage of disease-causing variants ranked as first out of the 134 exomes tested.

b LIRICAL was chosen as the reference, being the best performing VP software tool at ranking the disease-causing variants overall.

## Highlights

Next-generation sequencing technologies have made achieving a molecular diagnosis for a rare genetic disorder more and more feasible and, in turn, have enabled a more personalised clinical management of the affected patients and their families.

Identifying the one or two variants that are responsible for a certain disease phenotype from the millions identified by sequencing can be time-consuming and expensive.

Numerous phenotype-aware variant prioritisation (VP) software tools now exist to help semi-automate the molecular diagnosis process for rare diseases.

Although many of the published VP tools have many limitations, show a lack of maintenance, and become soon unfit for usage, several are up-to-date and demonstrate an impressive capacity in prioritising molecular diagnoses when tested on real patient data.

Adopting phenotype-aware VP software tools in diagnostics settings can efficiently assist the multidisciplinary teams of clinicians and scientists in reporting genetic diagnoses for rare disease.

## Outstanding questions

How can we tackle the overwhelming numbers of rare disease patients that undergo next-generation sequencing investigations and remain undiagnosed even after state-of-the-art data analysis?

How efficiently will the newly emerging functional variant assays be deployed at scale in clinical settings to clarify the precise link between disease-associated variants, gene function, and the full spectrum of phenotypes?

How can we strike a balance between the pressing need for sharing clinical genomic data and complying with patient data security on a global scale?

How efficiently will new algorithms be developed that can reliably prioritise noncoding and structural variants to make them available for further validation and molecular diagnosis at scale?

## Footnotes

i
https://github.com/exomiser/Exomiser/blob/master/README.md

ii
https://github.com/TheJacksonLaboratory/LIRICAL.git

iii
https://lirical.readthedocs.io/en/latest/

iv
https://github.com/phenopackets

v
www.ga4gh.org/

vi
https://web.stanford.edu/~xm24/Xrare/

vii
www.ncbi.nlm.nih.gov/clinvar
