# Supplementary material for: Phenotype-aware prioritisation of rare Mendelian disease variants
Source: Trends Genet. Author manuscript; Available in PMC 2023 Feb 24. (PMC9950798; doi:10.1016/j.tig.2022.07.002)
Supplement: Supplementary material [file NIHMS1872532-supplement-Supplementary_material.docx]

**Supplemental information**

**Phenotype-aware prioritisation of rare Mendelian disease variants**

Catherine Kelly^1^, Anita Szabo^2^, Nikolas Pontikos^2^, Gavin Arno^2^, Peter N. Robinson^3^, Jules O.B. Jacobsen^1^, Damian Smedley^1^, Valentina Cipriani^1,2,4,5^

^1^William Harvey Research Institute, Queen Mary University of London, London, EC1M 6BQ, UK; ^2^UCL Institute of Ophthalmology, University College London, London, EC1V 9EL, UK; ^3^The Jackson Laboratory for Genomic Medicine, Farmington, CT, 06032, USA; ^4^Moorfields Eye Hospital NHS Foundation Trust, London, EC1V 2PD, UK; ^5^UCL Genetics Institute, University College London, London, WC1E 6AA, UK

**Supplemental methods** 1

***Whole-exome sequencing (WES) data*** 1

***Software performance evaluation and statistical comparison using the IRD patient WES dataset*** 1

**Supplemental figures** 1

**Figure S1. Screenshots of some representative sections of the HTML output files for Exomiser (a) and LIRICAL (b) from the analysis of sample B240001.** 2

**Figure S2. Barplot (a) and boxplot (b) of the disease-causing variant ranking in the IRD patient WES dataset for the four successfully installed and run phenotype-aware VP software tools.** Variants filtered out/not prioritised (FO/NP) are not included in the boxplot analysis. 1

**Supplemental tables** 1

**Table S1. Characteristics of the inherited retinal disease (IRD) patient WES dataset with known molecular diagnosis and HPO-encoded clinical diagnosis.** 1

**Table S2. Molecular diagnosis (diagnosed gene, MOI/genotype and variant(s)) of the 134 patients in the IRD dataset.** 1

**References** 5

# **Supplemental methods**

# ***Whole-exome sequencing (WES) data***

Genomic DNA was purified from peripheral blood mononuclear cells for WES. WES was performed independently or as part of the National Institute for Health Research BioResource—Rare Disease study (NIHR BR-RD) as previously described [1]. The study protocols adhered to the tenets of the Declaration of Helsinki and received approval from the local ethics committee (MOOA1016, 13/EE/0325). Written informed consent was obtained from all participants, or their parents, before their inclusion in the study. Sequenced samples were analysed as part of the UCLex WES dataset using the Phenopolis bioinformatics pipeline [(Pontikos *et al.* 2017)](https://paperpile.com/c/fKZy7L/nkLP). Reads were aligned to the hg19 human reference sequence (build GRCh37) with NovoAlign (version 3.02.08; Novocraft Technologies, Petaling Jaya, Malaysia). The aligned reads were sorted by base pair position and duplicates were marked using NovoSort (version 1.03.05; Novocraft Technologies). Discordant reads were identified using SAMBLASTER (version 0.1.25) [2]. Variant calling and recalibration were performed using the Genome Analysis Tool Kit (GATK) (version 4.2.0.0) [(McKenna *et al.* 2010)](https://paperpile.com/c/fKZy7L/hhv3). As per the GATK Best Practices, variant calling from the aligned reads was performed using HaplotypeCaller and joint variant calling was performed using GenotypeGVCFs. The variants were filtered based on excess heterozygosity (ExcessHet > 54.69). Variant Quality Score Recalibration (VQSR) was performed separately for SNVs and indels to estimate the variant quality score log-odds (VQSLOD) and the FILTER values per variant. The individual steps of the variant calling and recalibration process were incorporated into a pipeline using nextflow (version 20.01.0).

# ***Software performance evaluation and statistical comparison using the IRD patient WES dataset***

For each individual in the IRD patient WES dataset, only those variants with a VCF FILTER value equal to ‘PASS’ were considered, either by enabling a corresponding option within the VP tool (available in Exomiser and PhenIX), or by filtering them out from the output list of prioritised variants (for Xrare). LIRICAL outputs only ‘PASS’ variants without the opportunity for the user to keep them. The ‘solved’ mode of inheritance (MOI) per each patient was leveraged as the inferred one in line with a previously published benchmarking on the same dataset [3]: only heterozygous variants were considered if the corresponding ‘solved’ MOI was autosomal dominant (AD), while only homozygous variants and/or at least two heterozygous variants per each gene were considered if the corresponding ‘solved’ MOIs were either autosomal recessive (AR) or X-linked recessive (XR) (Table S2). This was achieved either by enabling a corresponding option within the VP tool (available in Exomiser and PhenIX), or by processing the output lists of prioritised variants accordingly (for Xrare and LIRICAL).

We then assigned statistical ranks to the gene-variant observations contained in each individual’s processed output lists of prioritised variants based on the variant scoring from each VP tool. Equally scoring gene-variant observations (*ties*) were resolved by assigning the corresponding average rank. Finally, we noted the rank at which each VP tool outputted the known diagnosed disease variant(s) per each IRD patient. In the case of ‘solved’ compound heterozygous variants, the average rank was calculated. The following diagnostic measurements were calculated per each VP tool from confusion matrices for the correctly diagnosed variants matched in the 1st rank, up to the 5th rank and up to the 10th rank (using the total number of variants called in all 134 VCF files as the total number of actual positives and actual negatives): (i) *recall* (true positive rate) as true positives (TP)/actual positives; (ii) *precision* as TP/predicted positives.

To assess the pairwise agreement on the ranking results of the known disease-causing variants between different pairs of VP tools, we categorised the ranking results into five mutually exclusive disease-causing ranking bins: ‘Top’ (including top ties), ‘[2–6)’, ‘[6–11)’, ‘>=11’, and ‘Filtered out/Not prioritised’ (the latter being any disease-causing variant(s) failed to be kept in during the filtering/prioritisation step) and calculated the corresponding Cohen’s kappa coefficient (ƙ) (R module ‘fmsb’ version 0.7.3). Cohen’s kappa values were interpreted according to Landis and Koch’s guidelines (i.e., < 0.00 as “poor” agreement, 0.00–0.20 as “slight”, 0.21–0.40 as “fair”, 0.41–0.60 as “moderate”, 0.61–0.80 as “substantial”, and 0.81–1 as “almost perfect” agreement) [4]. The nonparametric Stuart–Maxwell test was then used to assess the marginal homogeneity of the five ranking bins simultaneously (R module ‘DescTools’ version 0.99.44). All the statistical analyses were conducted using R version 4.0.2 and Python 3.8.5 on the Queen Mary University (QMUL) Apocrita High-performance computing (HPC) facility (supported by QMUL Research-IT (http://doi.org/10.5281/zenodo.438045).

# **Supplemental figures**


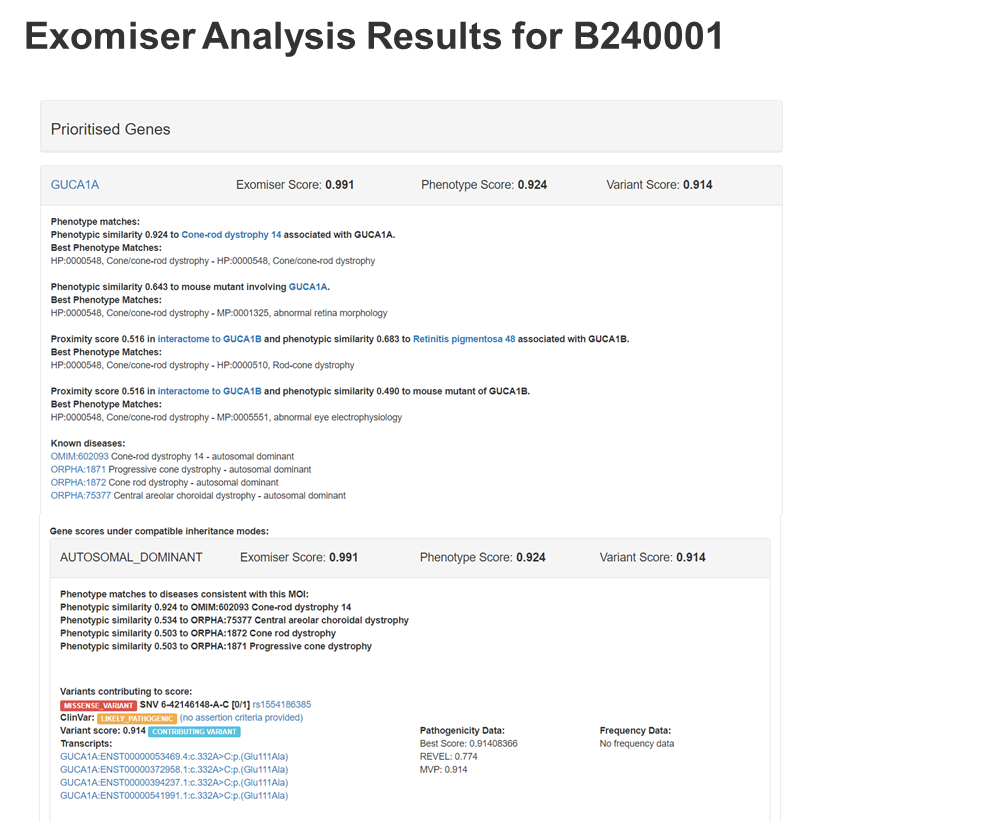


**a**


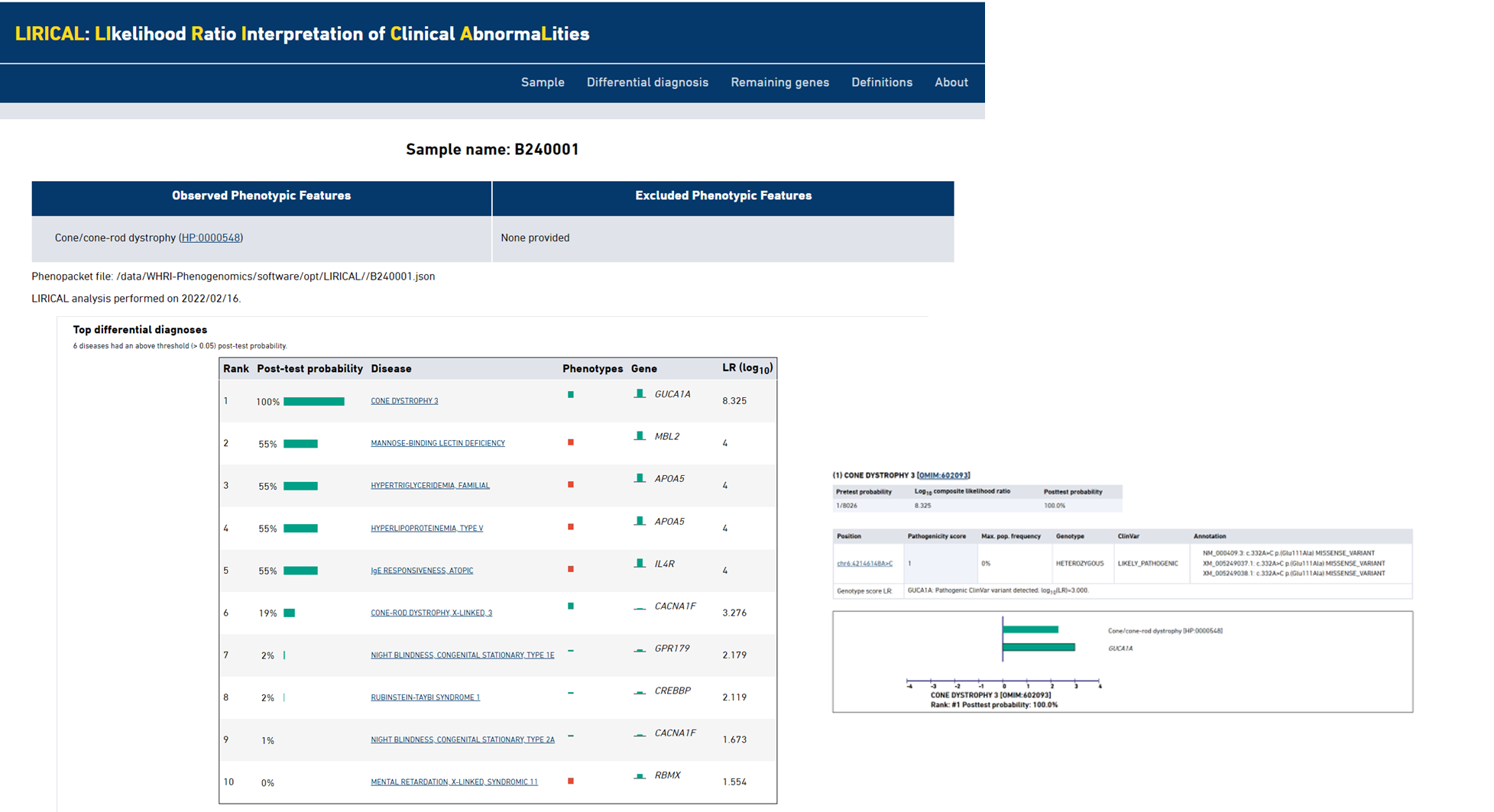


**b**

# **Figure S1. Screenshots of some representative sections of the HTML output files for Exomiser (a) and LIRICAL (b) from the analysis of sample B240001.**

**
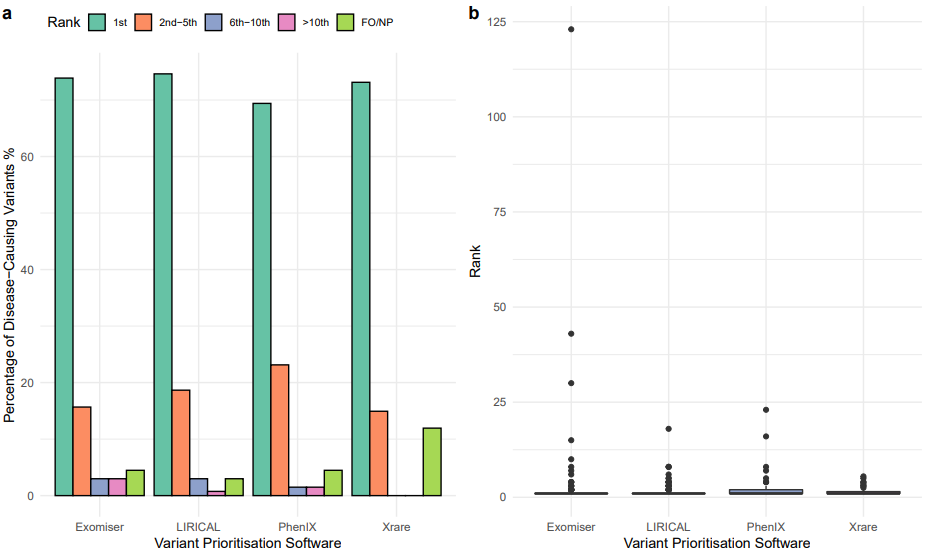
**

# **Figure S2. Barplot (a) and boxplot (b) of the disease-causing variant ranking in the IRD patient WES dataset for the four successfully installed and run phenotype-aware VP software tools.** Variants filtered out/not prioritised (FO/NP) are not included in the boxplot analysis.

#

# **Supplemental tables**

# **Table S1. Characteristics of the inherited retinal disease (IRD) patient WES dataset with known molecular diagnosis and HPO-encoded clinical diagnosis.**

| **Clinical diagnosis**  ***(HPO terms)*** | **Frequency (%)** |  | **Known disease-causing genotype** | **Frequency (%)** |  |  |
| --- | --- | --- | --- | --- | --- | --- |
| Retinitis pigmentosa  *(HP:0000510)* | 36 (26.9) |  | Homozygote | 72 (53.7) |  |  |
| Leber congenital amaurosis  *(HP:0000556, HP:0007758, HP:0000550, HP:0000639)* | 25 (18.7) |  | Compound  Heterozygote | 39 (29.1) |  |  |
| Macular dystrophy  *(HP:0007754)* | 16 (11.9) |  | Heterozygote | 13 (9.7) |  |  |
| Cone-rod dystrophy  *(HP:0000548)* | 14 (10.4) |  | Hemizygote | 10 (7.5) |  |  |
| Early onset retinal dystrophy  *(HP:0000556, HP:0011463)* | 9 (6.7) |  | *Total* | *134 (100)* |  |  |
| Usher syndrome type 2  *(HP:0000510, HP:0008527, HP:0012712, HP:0007642, HP:0000654)* | 8 (6.0) |  |  |  |  |  |
| Achromatopsia  *(HP:0011516, HP:0008275)* | 6 (4.5) |  | **Known disease-causing gene** | | | **Frequency** |
| Congenital stationary night blindness  *(HP:0007642, HP:0007984)* | 5 (3.7) |  | *USH2A* | | | 10 |
| Retinal dystrophy  *(HP:0000556)* | 3 (2.2) |  | *ABCA4, RPGR* | | | 8 |
| Stargardt's disease  *(HP:0007754)* | 2 (1.5) |  | *GUCY2D* | | | 7 |
| Usher syndrome type 1  *(HP:0000510, HP:0008527, HP:0011476, HP:0007642, HP:0001751, HP:0000654)* | 2 (1.5) |  | *BBS1, CNGB1, CRX* | | | 5 |
| Benign fleck retina  *(HP:0012045)* | 1 (0.7) |  | *CRB1, IMPG2* | | | 4 |
| Coloboma  *(HP:0000480)* | 1 (0.7) |  | *ADGRV1, AHI1, CERKL, LCA5, PDE6A, PROM1, RP1, TTLL5* | | | 3 |
| Familial exudative vitreoretinopathy  *(HP:0030490)* | 1 (0.7) |  | *ATF6, CDH3, CDHR1, CEP290, CFAP410, CNGB3, DRAM2, EYS, IQCB1, MFSD8, TULP1* | | | 2 |
| Foveal hypoplasia  *(HP:0007750)* | 1 (0.7) |  | *ADAM9, AGBL5, ARL2BP, CLN3, COL18A1, COL9A3, DHX38, GNAT2, GPR143, GPR179, GUCA1A, HPS6, IFT140, KCNJ13, KIF11, LRP5, MERTK, MYO7A, NMNAT1, NYX, PDE6B, PLA2G5, POC1B, RBP3, RP1L1, RPGRIP1, SPATA7, SRD5A3, TRNT1, TRPM1, TUB, YAP1* | | | 1 |
| Myopia and deafness (Stickler syndrome)  *(HP:0000545, HP:0008527)* | 1 (0.7) |  |  |  |  |  |
| Occult macular dystrophy  *(HP:0030636, HP:0030488)* | 1 (0.7) |  |  |  |  |  |
| Ocular albinism  *(HP:0001107, HP:0007730, HP:0007750, HP:0030464)* | 1 (0.7) |  |  |  |  |  |
| Optic atrophy  *(HP:0001138, HP:0000649, HP:0030487)* | 1 (0.7) |  |  |  |  |  |
| *Total* | *134 (100)* |  |  |  |  |  |

HPO = Human Phenotype Ontology

# **Table S2. Molecular diagnosis (diagnosed gene, MOI/genotype and variant(s)) of the 134 patients in the IRD dataset.**

| **Clinical**  **diagnosis** | **Patient**  **number** | **Gene** | **MOI/**  **Genotype** | **HGVS**  **variant 1** | **HGVS**  **variant 2** | **Total number of called variants in VCF file** | **VCF**  **FILTER**  **variant 1** | **VCF**  **FILTER**  **variant 2** |
| --- | --- | --- | --- | --- | --- | --- | --- | --- |
| Achromatopsia | P1 | *CNGB3* | AR/HOM | c.1810C>T:p.(Arg604*) |  | 76104 | PASS |  |
| Achromatopsia | P2 | *CNGB3* | AR/HOM | c.1148del:p.(Thr383Ilefs*13) |  | 64819 | PASS |  |
| Achromatopsia | P3 | *ATF6* | AR/HOM | c.970C>T:p.(Arg324Cys) |  | 39062 | PASS |  |
| Achromatopsia | P4 | *POC1B* | AR/HOM | c.130_138del:p.(Ile44_Leu46del) |  | 43403 | PASS |  |
| Achromatopsia | P5 | *ATF6* | AR/HOM | c.1187+5G>C:p.? |  | 45003 | PASS |  |
| Achromatopsia | P6 | *GNAT2* | AR/HOM | c.906C>A:p.(Tyr302*) |  | 58715 | PASS |  |
| Benign fleck retina | P7 | *PLA2G5* | AR/HOM | c.133G>T:p.(Gly45Cys) |  | 44031 | PASS |  |
| Coloboma | P8 | *YAP1* | AD/HET | c.284T>C:p.(Phe95Ser) |  | 85272 | PASS |  |
| Cone-rod dystrophy | P9 | *GUCA1A* | AD/HET | c.332A>C:p.(Glu111Ala) |  | 62625 | PASS |  |
| Cone-rod dystrophy | P10 | *AHI1* | AR/compHET | c.2945G>T:p.(Arg982Met) | c.1558A>G:p.(Lys520Glu) | 61770 | PASS | PASS |
| Cone-rod dystrophy | P11 | *PROM1* | AD/HET | c.1117C>T:p.(Arg373Cys) |  | 76256 | PASS |  |
| Cone-rod dystrophy | P12 | *PROM1* | AR/HOM | c.436C>T:p.(Arg146*) |  | 61092 | PASS |  |
| Cone-rod dystrophy | P13 | *RPGR* | XR/HEMI | c.3039_3040del:p.(Glu1018Argfs*60) |  | 62138 | Not called |  |
| Cone-rod dystrophy | P14 | *ABCA4* | AR/compHET | c.2861A>C:p.(Tyr954Ser) | c.3191-1G>T:p.? | 83535 | PASS | PASS |
| Cone-rod dystrophy | P15 | *MERTK* | AR/HOM | c.1470del:p.(Pro490Profs*25) |  | 84807 | PASS |  |
| Cone-rod dystrophy | P16 | *TTLL5* | AR/HOM | c.1627G>T:p.(Glu543*) |  | 81842 | PASS |  |
| Cone-rod dystrophy | P17 | *CERKL* | AR/HOM | c.316C>A:p.(Arg106Ser) |  | 80574 | PASS |  |
| Cone-rod dystrophy | P18 | *CFAP410* | AR/HOM | c.218G>C:p.(Arg73Pro) |  | 53447 | PASS |  |
| Cone-rod dystrophy | P19 | *AGBL5* | AR/HOM | c.323C>G:p.(Pro108Arg) |  | 84160 | PASS |  |
| Cone-rod dystrophy | P20 | *CFAP410* | AR/compHET | c.218G>C:p.(Arg73Pro) | c.655A>T:p.(Ile219Phe) | 84612 | PASS | PASS |
| Cone-rod dystrophy | P21 | *CDHR1* | AR/HOM | c.1463del:p.(Gly488Alafs*20) |  | 72791 | PASS |  |
| Cone-rod dystrophy | P22 | *CDHR1* | AR/HOM | c.1463del:p.(Gly488Alafs*20) |  | 74867 | PASS |  |
| Congenital stationary night blindness | P23 | *NYX* | XR/HEMI | c.998_1003del:p.(Leu333_Phe334del) |  | 64107 | PASS |  |
| Congenital stationary night blindness | P24 | *TRPM1* | AR/compHET | c.380G>A:p.(Gly127Glu) | c.832G>A:p.(Gly278Arg) | 62853 | PASS | PASS |
| Congenital stationary night blindness | P25 | *GPR179* | AR/compHET | c.870dup:p.(Asn291*) | c.1368del:p.(Phe456Leufs*30) | 83878 | PASS | PASS |
| Congenital stationary night blindness | P26 | *RBP3* | AR/HOM | c.3454G>T:p.(Glu1152*) |  | 83763 | PASS |  |
| Congenital stationary night blindness | P27 | *TRNT1* | AR/HOM | c.295C>T:p.(Arg99Trp) |  | 76777 | PASS |  |
| Early onset retinal dystrophy | P28 | *LCA5* | AR/HOM | c.617T>C:p.(Leu206Pro) |  | 80077 | PASS |  |
| Early onset retinal dystrophy | P29 | *PDE6A* | AR/HOM | c.769C>T:p.(Arg257*) |  | 82501 | PASS |  |
| Early onset retinal dystrophy | P30 | *BBS1* | AR/HOM | c.200G>A:p.(Arg67Glnext*-67) |  | 86820 | PASS |  |
| Early onset retinal dystrophy | P31 | *CDH3* | AR/HOM | c.2357del:p.(Gly786Alafs*7) |  | 84872 | PASS |  |
| Early onset retinal dystrophy | P32 | *CRX* | AD/HET | c.624T>A:p.(Tyr208*) |  | 83518 | PASS |  |
| Early onset retinal dystrophy | P33 | *IMPG2* | AR/HOM | c.1875_1879dup:p.(Pro627Leufs*25) |  | 84427 | PASS |  |
| Early onset retinal dystrophy | P34 | *ADAM9* | AR/HOM | c.967del:p.(Ser323Glnfs*33) |  | 82368 | PASS |  |
| Early onset retinal dystrophy | P35 | *IQCB1* | AR/compHET | c.825_828del:p.(Arg275Serfs*6) | c.745A>T:p.(Arg249*) | 88299 | PASS | PASS |
| Early onset retinal dystrophy | P36 | *CEP290* | AR/HOM | c.2450T>G:p.(Ile817Ser) |  | 60081 | PASS |  |
| Familial exudative vitreoretinopathy | P37 | *LRP5* | AR/compHET | c.1435G>A:p.(Gly479Arg) | c.4097A>G:p.(Asp1366Gly) | 41763 | PASS | PASS |
| Foveal hypoplasia | P38 | *HPS6* | AR/HOM | c.779G>A:p.(Gly260Glu) |  | 84154 | PASS |  |
| Leber congenital amaurosis | P39 | *ABCA4* | AR/compHET | c.4918C>T:p.(Arg1640Trp) | c.2041C>T:p.(Arg681*) | 63663 | PASS | PASS |
| Leber congenital amaurosis | P40 | *GUCY2D* | AR/HOM | c.2120T>C:p.(Leu707Pro) |  | 84506 | PASS |  |
| Leber congenital amaurosis | P41 | *COL18A1* | AR/HOM | c.714dup:p.(Gly239Argfs*9) |  | 82623 | PASS |  |
| Leber congenital amaurosis | P42 | *GUCY2D* | AR/HOM | c.652del:p.(Met218Trpfs*13) |  | 81906 | PASS |  |
| Leber congenital amaurosis | P43 | *DHX38* | AR/HOM | c.971G>A:p.(Arg324Gln) |  | 81937 | PASS |  |
| Leber congenital amaurosis | P44 | *SRD5A3* | AR/HOM | c.57G>A:p.(Trp19*) |  | 83633 | PASS |  |
| Leber congenital amaurosis | P45 | *GUCY2D* | AR/HOM | c.2836G>A:p.(Ala946Thr) |  | 80812 | PASS |  |
| Leber congenital amaurosis | P46 | *RPGRIP1* | AR/compHET | c.711del:p.(Pro237Profs*38) | c.2786A>G:p.(Tyr929Cys) | 79280 | PASS | PASS |
| Leber congenital amaurosis | P47 | *CEP290* | AR/HOM | c.148C>T:p.(His50Tyr) |  | 81193 | PASS |  |
| Leber congenital amaurosis | P48 | *CRB1* | AR/compHET | c.976T>C:p.(Cys326Arg) | c.1798C>T:p.(Gln600*) | 47304 | PASS | PASS |
| Leber congenital amaurosis | P49 | *NMNAT1* | AR/HOM | c.53A>G:p.(Asn18Ser) |  | 37059 | PASS |  |
| Leber congenital amaurosis | P50 | *CLN3* | AR/HOM | c.932C>A:p.(Ser311Tyr) |  | 39008 | PASS |  |
| Leber congenital amaurosis | P51 | *TUB* | AR/HOM | c.1194_1195del:p.(Arg398Serfs*10) |  | 39122 | PASS |  |
| Leber congenital amaurosis | P52 | *KCNJ13* | AR/HOM | c.496C>T:p.(Arg166*) |  | 42064 | PASS |  |
| Leber congenital amaurosis | P53 | *LCA5* | AR/HOM | c.1676C>A:p.(Ser559*) |  | 43060 | PASS |  |
| Leber congenital amaurosis | P54 | *RP1* | AR/HOM | c.1458_1461dup:p.(Glu488*) |  | 41582 | PASS |  |
| Leber congenital amaurosis | P55 | *BBS1* | AR/HOM | c.200G>A:p.(Arg67Glnext*-67) |  | 41242 | PASS |  |
| Leber congenital amaurosis | P56 | *IMPG2* | AR/HOM | c.68dup:p.(Asp23Glufs*29) |  | 84163 | Non-PASS^a^ |  |
| Leber congenital amaurosis | P57 | *IFT140* | AR/HOM | c.1451C>T:p.(Thr484Met) |  | 84971 | PASS |  |
| Leber congenital amaurosis | P58 | *SPATA7* | AR/HOM | c.864dup:p.(Pro289Thrfs*6) |  | 83294 | PASS |  |
| Leber congenital amaurosis | P59 | *GUCY2D* | AR/HOM | c.2395_2398dup:p.(His800Argfs*20) |  | 85060 | PASS |  |
| Leber congenital amaurosis | P60 | *TULP1* | AR/HOM | c.751G>T:p.(Glu251*) |  | 88629 | PASS |  |
| Leber congenital amaurosis | P61 | *IQCB1* | AR/HOM | c.1363C>T:p.(Arg455*) |  | 81580 | PASS |  |
| Leber congenital amaurosis | P62 | *ABCA4* | AR/compHET | c.161G>A:p.(Cys54Tyr) | c.2160+1G>C:p.? | 63216 | PASS | PASS |
| Leber congenital amaurosis | P63 | *GUCY2D* | AR/HOM | c.2828dup:p.(Arg944Alafs*27) |  | 76562 | PASS |  |
| Macular dystrophy | P64 | *MFSD8* | AR/HOM | c.1361T>C:p.(Met454Thr) |  | 80887 | PASS |  |
| Macular dystrophy | P65 | *CRX* | AD/HET | c.774T>A:p.(Tyr258*) |  | 97989 | PASS |  |
| Macular dystrophy | P66 | *GUCY2D* | AD/HET | c.2512C>T:p.(Arg838Cys) |  | 78574 | PASS |  |
| Macular dystrophy | P67 | *GUCY2D* | AD/HET | c.2512C>T:p.(Arg838Cys) |  | 83218 | PASS |  |
| Macular dystrophy | P68 | *CRX* | AD/HET | c.121C>T:p.(Arg41Trp) |  | 58443 | PASS |  |
| Macular dystrophy | P69 | *RPGR* | XR/HEMI | c.3317dup:p.(Ser1107Valfs*4) |  | 53294 | PASS |  |
| Macular dystrophy | P70 | *CRX* | AD/HET | c.127C>T:p.(Arg43Cys) |  | 53199 | PASS |  |
| Macular dystrophy | P71 | *ABCA4* | AR/compHET | c.3522G>A:p.(=) | c.5527C>G:p.(Arg1843Gly) | 54492 | PASS | PASS |
| Macular dystrophy | P72 | *CRX* | AD/HET | c.272G>A:p.(Arg91Lys) |  | 65684 | PASS |  |
| Macular dystrophy | P73 | *ABCA4* | AR/compHET | c.5882G>A:p.(Gly1961Glu) | c.885del:p.(Asp295Aspfs*5) | 54616 | PASS | PASS |
| Macular dystrophy | P74 | *DRAM2* | AR/HOM | c.362A>T:p.(His121Leu) |  | 55008 | PASS |  |
| Macular dystrophy | P75 | *TTLL5* | AR/HOM | c.1586_1589del:p.(Glu529Valfs*2) |  | 54306 | PASS |  |
| Macular dystrophy | P76 | *RPGR* | XR/HEMI | c.3178_3179del:p.(Glu1066Glyfs*12) |  | 53423 | PASS |  |
| Macular dystrophy | P77 | *CDH3* | AR/HOM | c.1568del:p.(Asn523Metfs*14) |  | 54480 | PASS |  |
| Macular dystrophy | P78 | *TTLL5* | AR/compHET | c.401del:p.(Leu134Argfs*45) | c.3354G>A:p.(Trp1118*) | 53563 | PASS | PASS |
| Macular dystrophy | P79 | *DRAM2* | AR/compHET | c.217_225del:p.(Val73_Tyr75del) | c.79T>C:p.(Tyr27His) | 54037 | PASS | PASS |
| Myopia and deafness  (Stickler syndrome) | P80 | *COL9A3* | AR/HOM | c.1739dup:p.(Gly581Trpfs*20) |  | 95813 | PASS |  |
| Occult macular dystrophy | P81 | *MFSD8* | AR/compHET | c.103C>T:p.(Arg35*) | c.1006G>C:p.(Glu336Gln) | 56266 | PASS | PASS |
| Ocular albinism | P82 | *GPR143* | XR/HEMI | c.839A>G:p.(Asn280Ser) |  | 70118 | PASS |  |
| Optic atrophy | P83 | *KIF11* | AD/HET | c.247C>T:p.(Arg83*) |  | 77429 | PASS |  |
| Retinal dystrophy | P84 | *EYS* | AR/compHET | c.7994G>A:p.(Gly2665Glu) | c.2976T>A:p.(Cys992*) | 62067 | PASS | PASS |
| Retinal dystrophy | P85 | *LCA5* | AR/HOM | c.633_639del:p.(Glu211Aspfs*13) |  | 64189 | PASS |  |
| Retinal dystrophy | P86 | *CERKL* | AR/HOM | c.316C>A:p.(Arg106Ser) |  | 63468 | PASS |  |
| Retinitis pigmentosa | P87 | *PDE6A* | AR/HOM | c.1630C>T:p.(Arg544Trp) |  | 62353 | PASS |  |
| Retinitis pigmentosa | P88 | *RPGR* | XR/HEMI | c.126T>G:p.(Cys42Trp) |  | 64950 | PASS |  |
| Retinitis pigmentosa | P89 | *USH2A* | AR/compHET | c.2299del:p.(Glu767Serfs*21) | c.6050-1G>A:p.? | 61785 | PASS | PASS |
| Retinitis pigmentosa | P90 | *USH2A* | AR/compHET | c.7334C>T:p.(Ser2445Phe) | c.3902G>T:p.(Gly1301Val) | 61257 | PASS | PASS |
| Retinitis pigmentosa | P91 | *IMPG2* | AR/compHET | c.2426G>A:p.(Trp809*) | c.3412_3413insAA:p.(Ser1138Lysfs*21) | 58900 | PASS | PASS |
| Retinitis pigmentosa | P92 | *CNGB1* | AR/compHET | c.952C>T:p.(Gln318*) | c.3A>T:p.0? | 58441 | PASS | PASS |
| Retinitis pigmentosa | P93 | *PDE6A* | AR/compHET | c.1630C>T:p.(Arg544Trp) | c.769C>T:p.(Arg257*) | 62783 | PASS | PASS |
| Retinitis pigmentosa | P94 | *RPGR* | XR/HEMI | c.2323_2324del:p.(Arg775Glufs*59) |  | 62413 | PASS |  |
| Retinitis pigmentosa | P95 | *CERKL* | AR/HOM | c.847C>T:p.(Arg283*) |  | 62168 | PASS |  |
| Retinitis pigmentosa | P96 | *IMPG2* | AR/compHET | c.2426G>A:p.(Trp809*) | c.118G>T:p.(Glu40*) | 61709 | PASS | PASS |
| Retinitis pigmentosa | P97 | *RPGR* | XR/HEMI | c.2236_2237del:p.(Lys751Glyfs*18) |  | 65060 | PASS |  |
| Retinitis pigmentosa | P98 | *RP1* | AD/HET | c.2206dup:p.(Thr736Asnfs*4) |  | 62701 | PASS |  |
| Retinitis pigmentosa | P99 | *BBS1* | AR/HOM | c.1169T>G:p.(Met390Arg) |  | 61922 | PASS |  |
| Retinitis pigmentosa | P100 | *RPGR* | XR/HEMI | c.914dup:p.(Asn305Lysfs*41) |  | 63799 | PASS |  |
| Retinitis pigmentosa | P101 | *CRB1* | AR/HOM | c.1337T>C:p.(Ile446Thr) |  | 63117 | PASS |  |
| Retinitis pigmentosa | P102 | *RP1* | AR/HOM | c.5883del:p.(Gln1961Glnfs*16) |  | 60611 | PASS |  |
| Retinitis pigmentosa | P103 | *CNGB1* | AR/compHET | c.664C>T:p.(Gln222*) | c.262C>T:p.(Gln88*) | 62516 | PASS | PASS |
| Retinitis pigmentosa | P104 | *BBS1* | AR/HOM | c.1169T>G:p.(Met390Arg) |  | 62360 | PASS |  |
| Retinitis pigmentosa | P105 | *USH2A* | AR/compHET | c.9882C>G:p.(Cys3294Trp) | c.653T>A:p.(Val218Glu) | 63669 | PASS | PASS |
| Retinitis pigmentosa | P106 | *USH2A* | AR/compHET | c.13274C>T:p.(Thr4425Met) | c.8981G>A:p.(Trp2994*) | 63493 | PASS | PASS |
| Retinitis pigmentosa | P107 | *CRB1* | AR/HOM | c.782A>G:p.(Asn261Ser) |  | 60892 | PASS |  |
| Retinitis pigmentosa | P108 | *RPGR* | XR/HEMI | c.2250_2251del:p.(Lys751Glyfs*18) |  | 64016 | PASS |  |
| Retinitis pigmentosa | P109 | *AHI1* | AR/compHET | c.2429C>T:p.(Pro810Leu) | c.2087A>G:p.(His696Arg) | 64897 | PASS | PASS |
| Retinitis pigmentosa | P110 | *EYS* | AR/HOM | c.490C>T:p.(Arg164*) |  | 58814 | PASS |  |
| Retinitis pigmentosa | P111 | *CRB1* | AR/HOM | c.1819G>T:p.(Gly607*) |  | 61649 | PASS |  |
| Retinitis pigmentosa | P112 | *CNGB1* | AR/compHET | c.3139_3142dup:p.(Ala1048Glyfs*13) | c.3A>T:p.0? | 63280 | PASS | PASS |
| Retinitis pigmentosa | P113 | *CNGB1* | AR/HOM | c.3A>T:p.0? |  | 62220 | PASS |  |
| Retinitis pigmentosa | P114 | *BBS1* | AR/HOM | c.200G>A:p.(Arg67Glnext*-67) |  | 61218 | PASS |  |
| Retinitis pigmentosa | P115 | *PDE6B* | AR/HOM | c.1485dup:p.(Pro496Alafs*5) |  | 62917 | PASS |  |
| Retinitis pigmentosa | P116 | *CNGB1* | AR/HOM | c.761+2T>A:p.? |  | 61547 | PASS |  |
| Retinitis pigmentosa | P117 | *AHI1* | AR/compHET | c.2090C>T:p.(Pro697Leu) | c.660del:p.(Pro220Profs*11) | 83552 | PASS | PASS |
| Retinitis pigmentosa | P118 | *RP1L1* | AR/HOM | c.603del:p.(Gly201Glyfs*30) |  | 42386 | PASS |  |
| Retinitis pigmentosa | P119 | *ARL2BP* | AR/HOM | c.134T>G:p.(Met45Arg) |  | 80025 | PASS |  |
| Retinitis pigmentosa | P120 | *ABCA4* | AR/HOM | c.3393del:p.(Ala1131Alafs*17) |  | 67644 | PASS |  |
| Retinitis pigmentosa | P121 | *PROM1* | AR/HOM | c.2346del:p.(Phe782Phefs*10) |  | 60795 | PASS |  |
| Retinitis pigmentosa | P122 | *TULP1* | AR/compHET | c.1081C>T:p.(Arg361*) | c.1255C>T:p.(Arg419Trp) | 75595 | non-PASS^b^ | PASS |
| Stargardt disease | P123 | *ABCA4* | AR/HOM | c.6658C>T:p.(Gln2220*) |  | 62735 | PASS |  |
| Stargardt disease | P124 | *ABCA4* | AR/compHET | c.3364G>A:p.(Glu1122Lys) | c.1906C>T:p.(Gln636*) | 62747 | PASS | PASS |
| Usher syndrome type I | P125 | *MYO7A* | AR/compHET | c.598A>G:p.(Ser200Gly) | c.611A>C:p.(Lys204Thr) | 52565 | PASS | PASS |
| Usher syndrome type I | P126 | *ADGRV1* | AR/compHET | c.13919G>A:p.(Gly4640Glu) | c.18610G>A:p.(Gly6204Ser) | 58256 | PASS | PASS |
| Usher syndrome type II | P127 | *USH2A* | AR/compHET | c.3832_3834del:p.(Leu1278del) | c.920_923dup:p.(His308Glnfs*16) | 61025 | PASS | Non-PASS^a^ |
| Usher syndrome type II | P128 | *USH2A* | AR/HOM | c.2299del:p.(Glu767Serfs*21) |  | 60439 | PASS |  |
| Usher syndrome type II | P129 | *USH2A* | AR/compHET | c.1055C>T:p.(Thr352Ile) | c.12819T>A:p.(Tyr4273*) | 63746 | PASS | PASS |
| Usher syndrome type II | P130 | *ADGRV1* | AD/HET | c.8807C>G:p.(Ser2936*) |  | 62624 | PASS |  |
| Usher syndrome type II | P131 | *USH2A* | AR/compHET | c.12954C>A:p.(Tyr4318*) | c.5603T>G:p.(Phe1868Cys) | 64785 | PASS | PASS |
| Usher syndrome type II | P132 | *USH2A* | AR/compHET | c.7594+2T>A:p.? | c.4632G>C:p.(Lys1544Asn) | 85590 | PASS | PASS |
| Usher syndrome type II | P133 | *ADGRV1* | AR/compHET | c.3290-1G>A:p.? | c.11242dup:p.(Glu3748Glyfs*10) | 53331 | PASS | PASS |
| Usher syndrome type II | P134 | *USH2A* | AR/compHET | c.5012G>A:p.(Gly1671Asp) | c.5252G>T:p.(Gly1751Val) | 55402 | PASS | PASS |

Clinical diagnosis as assigned by a consultant ophthalmologist before performing whole-exome sequencing.

MOI = Mode of Inheritance; HGVS = Human Genome Variation Society; VCF = Variant Call Format; HET = heterozygote; HOM = homozygote; compHET = compound heterozygote; HEMI = hemizygote.

^a^ VQSRTrancheINDEL99.00to99.50

^b^ VQSRTrancheSNP99.80to99.90

# **References**

1. Carss, K.J. *et al.* (2017) Comprehensive Rare Variant Analysis via Whole-Genome Sequencing to Determine the Molecular Pathology of Inherited Retinal Disease. *Am. J. Hum. Genet.* 100, 75-90. 10.1016/j.ajhg.2016.12.003

2. Faust, G.G. and Hall, I.M. (2014) SAMBLASTER: fast duplicate marking and structural variant read extraction. *Bioinformatics* 30, 2503-2505. 10.1093/bioinformatics/btu314

3. Cipriani, V. *et al.* (2020) An Improved Phenotype-Driven Tool for Rare Mendelian Variant Prioritization: Benchmarking Exomiser on Real Patient Whole-Exome Data. *Genes* 11. 10.3390/genes11040460

4. Landis, J.R. and Koch, G.G. (1977) The measurement of observer agreement for categorical data. *Biometrics* 33, 159-174
